# Robotic goalie with 3 ms reaction time at 4% CPU load using event-based dynamic vision sensor

**DOI:** 10.3389/fnins.2013.00223

**Published:** 2013-11-21

**Authors:** Tobi Delbruck, Manuel Lang

**Affiliations:** Department of Information Technology and Electrical Engineering, Institute of Neuroinformatics, UNI-ETH ZurichZurich, Switzerland

**Keywords:** asynchronous vision sensor, address-event representation, AER, high-speed visually guided robotics, high frame rate, neuromorphic system, soccer

## Abstract

Conventional vision-based robotic systems that must operate quickly require high video frame rates and consequently high computational costs. Visual response latencies are lower-bound by the frame period, e.g., 20 ms for 50 Hz frame rate. This paper shows how an asynchronous neuromorphic dynamic vision sensor (DVS) silicon retina is used to build a fast self-calibrating robotic goalie, which offers high update rates and low latency at low CPU load. Independent and asynchronous per pixel illumination change events from the DVS signify moving objects and are used in software to track multiple balls. Motor actions to block the most “threatening” ball are based on measured ball positions and velocities. The goalie also sees its single-axis goalie arm and calibrates the motor output map during idle periods so that it can plan open-loop arm movements to desired visual locations. Blocking capability is about 80% for balls shot from 1 m from the goal even with the fastest-shots, and approaches 100% accuracy when the ball does not beat the limits of the servo motor to move the arm to the necessary position in time. Running with standard USB buses under a standard preemptive multitasking operating system (Windows), the goalie robot achieves median update rates of 550 Hz, with latencies of 2.2 ± 2 ms from ball movement to motor command at a peak CPU load of less than 4%. Practical observations and measurements of USB device latency are provided^1^.

## Introduction

The notion of a “frame” of video data is embedded in machine vision. High speed frame-based vision is expensive because it is based on a series of pictures taken at a constant rate. The pixels are sampled repetitively even if their values are unchanged. Short-latency vision problems require high frame rate and produce massive amount of input data. At high frame rate, few CPU instructions are available for processing each pixel. For example, a VGA 640 × 480 pixel image sensor at 1 kHz frame rate delivers data at a rate of 307 M pixels/s, or a pixel every 3.3 ns. At usable instruction rates of 1 GHz a computer would only be able to dedicate 3 instructions per pixel to processing this information. This high data rate, besides requiring specialized computer interfaces and cabling (Wilson, 2007), makes it expensive in terms of power to deal with the data, especially in real time or embedded devices. Specialized high-frame-rate machine vision cameras with region of interest (ROI) or binning (sub-sampling) capabilities can reduce the amount of data significantly, but the ROI and binning must be controlled by software and the ROI is limited to a single region, reducing its usefulness for tracking multiple objects. Tracking a single object requires steering the ROI to follow the object. The latency of this ROI control must be kept short to avoid losing the object and ROI control can become quite complex to implement. Ref. (Graetzel et al., 2006), for example, describes a fruit-fly wing-beat analyzer that uses Kalman filtering to move the ROI in anticipation of where it should be according to the Kalman filter parameters, and even to time-multiplex the ROI between different parts of the scene. The computer must process all the pixels for each ROI or binned frame of data and ROI control latencies must be kept short if the object motion is not predictable.

By contrast, in the camera used for this paper, data are generated and transmitted asynchronously only from pixels with changing brightness. In a situation where the camera is fixed and the illumination is not varying only moving objects generate events. This situation reduces the delay compared to waiting for and processing an entire frame. Also, processor power consumption is related to the scene activity and can be reduced by shorter processing time and longer processor sleep phases between processing cycles.

This paper describes the results of experiments in low-latency visual robotics using an asynchronous dynamic vision sensor (DVS) (Lichtsteiner et al., 2006, 2007) as the input sensor, a standard PC as the processor, standard USB interfaces, and a standard hobby servo motor as the output.

Specifically, this paper demonstrates that independent pixel event data of a DVS are well-suited for object tracking and real-time visual feedback control. The simple but highly efficient object-tracking algorithm is implemented on a general purpose CPU. The experiments show that such a robot, although based on traditional, cheap, ubiquitous PC components like USB and a standard preemptive operating system (Windows) a simple programmable Java control application achieves reaction times on par with high speed conventional machine vision hardware running on dedicated real-time operating systems consuming the resources of an entire computer.

This paper expands on a brief conference report (Delbruck and Lichtsteiner, 2007) by including the new feature of self-calibration, more detailed descriptions of the algorithms, and new measurements of performance and latency particularly relating to USB interfaces. Other related work that has integrated an event-based neuromorphic vision sensor in a robot includes CAVIAR, a completely spike-hardware based visual tracking system (Serrano-Gotarredona et al., 2009), a pencil balancing robot using a pair of embedded-processor DVS cameras (Conradt et al., 2009a), which was first prototyped using two DVS cameras interfaced by USB (Conradt et al., 2009b), a demonstration of real-time stereo distance estimation computed on an FPGA with 2 DVS cameras (Domínguez-Morales et al., 2012), an embedded FPGA-based visual feedback system using a DVS (Linares-Barranco et al., 2007), and a micro gripper haptic feedback system (Ni et al., 2013) which uses a DVS as one of the two input sensors.

## Materials and methods: goalie architecture

**Figure 1:**
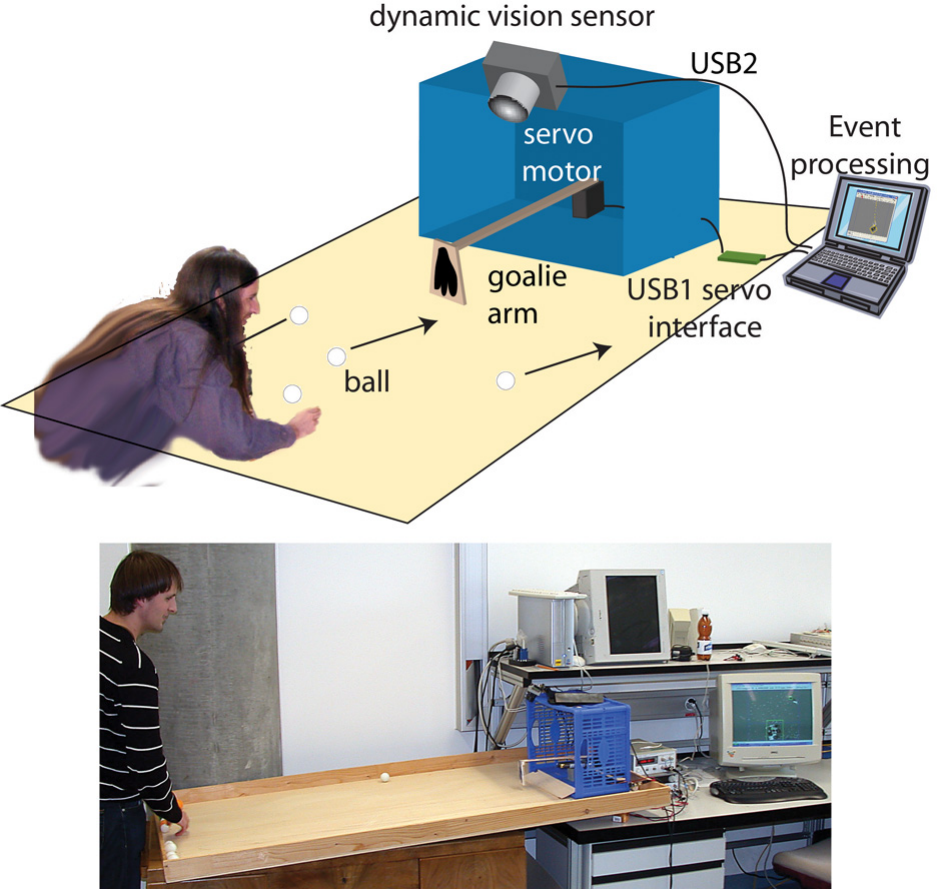
**Goalie robot illustration and a photo of the setup, showing the placement of vision sensor, goalie arm, and goal.** The white or orange balls have a diameter of 3 or 4 cm and are viewed against the light brown wood table. The reflectance ratio between balls and table is about 1.3. The retina view extends out to 1 m from the goal line. The goalie hand is 5 cm wide and the goal is 45 cm wide.

The application presented here is a self-calibrating soccer goalie robot (Figure 1). The robotic goalie blocks balls shot at a goal using a single-axis arm with only a single degree of freedom. Figure 1 shows our goalie robot hardware architecture. Players attempt to score by shooting balls at the goal (either by rolling or flicking with their fingernails) and the goalie robot tries to block all balls from entering the goal. Only balls that roll or slide along or near the table surface can be blocked and this limitation is what enables the solution to the blocking problem without stereo vision or some other means of determining the height of the ball over the table. The fact that the balls move along the surface of the table means that their 3D position can (implicitly in this application) be determined from the ball's 2D image position. The goalie is self-calibrating i.e., by visual observation it learns the motor control to arm position relationship. When turned on the goalie is one of 4 distinct states. In the *active* state, the goalie has determined that a ball is approaching the goal that can be blocked and tries to block it. Between balls, the goalie is *relaxed* to the middle position. When no definite balls have been seen for a few seconds, the goalie enters *sleeping* state where it does not respond to every movement in the scene. This state reduces apparently spastic movements in response to people walking by, hands, etc. After several minutes in sleeping state the goalie enters the *learning* in which it recalibrates itself. The goalie wakes up from *sleeping* to become *active* when it again sees a definite ball.

The rest of this section will describe the individual components of the system.

### Dynamic vision sensor

**Figure 2:**
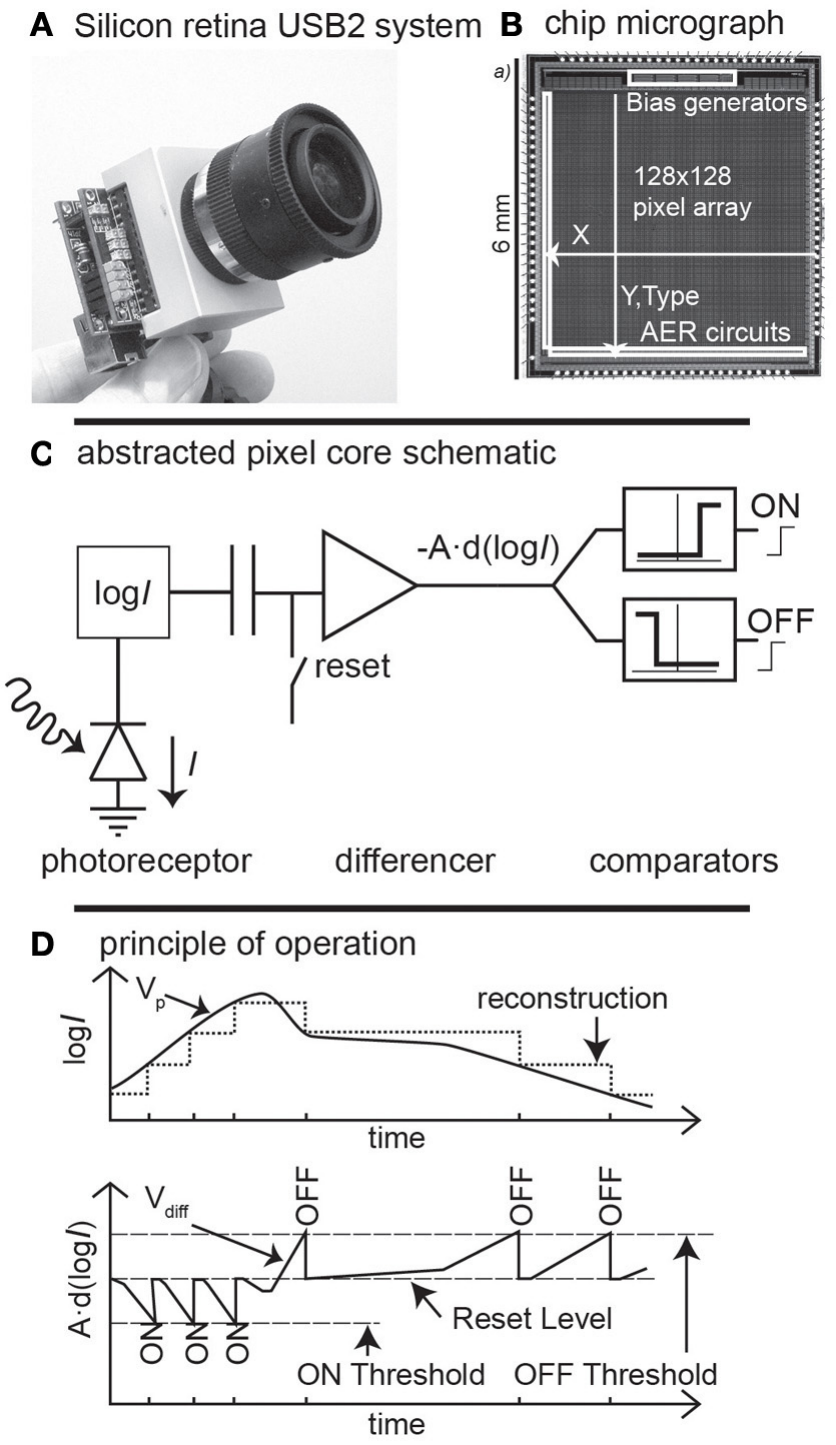
**Characteristics of the dynamic vision sensor (Tmpdiff128). (A)** the dynamic vision sensor with its lens and USB2.0 interface. **(B)** A die photograph. Pixels generate address-events, with the address formed from the *x, y*, location and ON or OFF type **(C)** an abstracted schematic of the pixel, which responds with events to fixed-size changes of log intensity. **(D)** How the ON and OFF events are internally represented and output in response to an input signal. Figure adapted from Lichtsteiner et al. (2006).

Conventional image sensors see the world as a sequence of frames, each consisting of many pixels. In contrast, the DVS is an example of a sensor that outputs digital address events (spikes) in response of temporal contrast at the moments that pixels see changing intensity (Lichtsteiner et al., 2006, 2007; Delbruck et al., 2010) (Figure 2). Like an abstraction of some classes of retinal ganglion cell spikes seen in biology, each event that is output from the DVS indicates that the log intensity at a pixel has changed by an amount *T* since the last event. *T* is a global event threshold which is typically set to about 15% contrast in this goalie robot application. In contrast to biology, the serial data path used requires the events to carry address information of what pixels number has changed. The address encodes the positive or negative brightness changes (ON or OFF) with one bit and the rest of the bits encode the row and column addresses of the triggering pixel. This representation of “change in log intensity” encodes scene reflectance change, as long as the illumination is constant over time, but not necessarily over space. Because this computation is based on a compressive logarithmic transformation in each pixel, it also allows for wide dynamic range operation (120 dB, compared with e.g., 60 dB for a high quality traditional image sensor).

This neuromorphic abstraction of the transient pathway seen in biology turns out to be useful for a number of reasons. The wide dynamic range means that the sensor can be used with uncontrolled natural lighting, even when the scene illumination is non-uniform and includes strong shadows, as long as they are not moving. The asynchronous response property also means that the events have the timing precision of the pixel response rather than being quantized to the traditional frame rate. Thus, the “effective frame rate” is typically several kHz and is set by the available illumination which determines the pixel bandwidth. The temporal redundancy reduction reduces the output data rate for scenes in which most pixels are not changing. The design of the pixel also allows for uniformity of response: the mismatch between pixel contrast thresholds is 2.1% contrast and the event threshold can be set down to 10% contrast, allowing the device to sense real-world contrast signals rather than only artificial high contrast stimuli. The vision sensor has integrated digitally controlled biases that minimize chip-to-chip variation in parameters and temperature sensitivity. Equipped with an USB2.0 high-speed interface, the DVS camera delivers the time-stamped address-event representation (**AER**) address-events to a host PC with timestamp resolution of 1 us.

### Event-driven tracking algorithm

Events from the DVS are processed inside jAER, an open-source Java software infrastructure for processing event-based sensor outputs (2007). The goalie implementation consists of about 3 k non-comment lines of code. The goalie software implementation is open-sourced in jAER.

**Figure 3:**
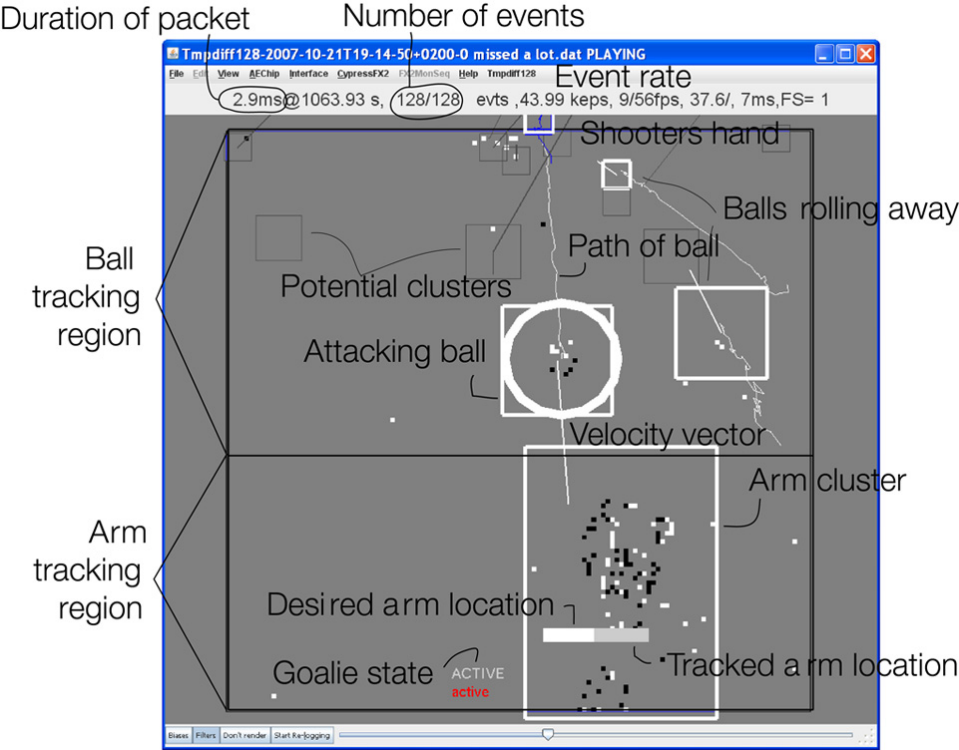
**Snapshot of action showing 128 events (all events within 2.9 ms) from the vision sensor.** It shows 5 tracked objects (the middle 3 are real balls, the top one is the shooter's hand, and the bottom object is the goalie arm). The Attacking ball rolling toward the goal (and being blocked) is marked with a circle; other balls are tracked but ignored. The thin squares represent potential clusters that have not received sufficient support. The velocity vectors of each ball are also shown as a slightly thicker line and have been computed by least squares linear regression over the past 10 packets of events. The goalie arm is being moved to the left bar and the presently tracked location of the arm is shown as a light bar inside the arm cluster. The state of the goalie is indicated as “active” meaning a tracked ball is being blocked. The balls generate average event rates of 3–30 keps (kilo events per second). The mean event rate for this packet was 44 keps.

The ball and arm tracker is an event-driven cluster tracker described briefly in (Lichtsteiner et al., 2006; Litzenberger et al., 2006) (Figure 3) and further enhanced in this work. This algorithm is inspired by the mean-shift approach used in frame-based vision (Cheng, 1995; Comaniciu and Ramesh, 2000). Each “cluster” models a moving object as a source of events. Visible clusters are indicated by the boxes in Figure 3. Events that fall within the cluster move the cluster position, and a cluster is only considered supported (“visible”) when it has received a threshold number of events. Clusters that lose support for a threshold period are pruned. Overlapping clusters are merged periodically at 1 ms intervals. Cluster positions are updated by using a mixing factor that mixes the old position with the new observations using fixed factors. Thus, the time constant governing cluster position is inversely proportional to the evidence (event rate).

The advantages of the cluster tracker are:

1. There is no frame correspondence problem because the events continuously update the cluster locations during the movement of the objects, and the faster the objects move, the more events they generate.
2. Only pixels that generate events need to be processed. The cost of this processing is dominated by the search for the nearest existing cluster, which is a cheap operation because there are only a few clusters.
3. Memory cost is low because there is no full frame memory, only cluster memory, and each cluster requires only a few hundred bytes of memory.

In the goalie application the objects have a known size and roll on a flat surface so tracked clusters have an image space radius determined by their perspective location in the scene.

The algorithm runs on each packet of combined events received from USB transmission, typically 128 (or fewer):

1. Pruning: Iterate over all existing clusters, pruning out those clusters that have not received sufficient support. A cluster is pruned if it has not received an event to support it within a given time, typically 10 ms in this application.
2. Merging: Iterate over all clusters to merge overlapping clusters. This merging operation is necessary because new clusters can be formed when an object grows larger as it approaches the vision sensor. For each cluster rectangle that overlaps the rectangle of another cluster, merge the two clusters into a new cluster and discard the previous clusters. The new cluster takes on the history of the older two clusters and its position is the weighted average of the locations of the source clusters. The averaging is weighted by the number of events in each source cluster. This weighting reduces the jitter in the cluster location caused by merging. This iteration continues as long as there are overlapping clusters.
3. Positioning: For each event, find the nearest cluster that contains the event. The predicted location of each cluster that is considered in this step is computed using its present cluster location combined with the present cluster velocity estimate and the time between this event and the last one that updated the cluster. This way, an event can be in a cluster's predicted location even if it is not inside the last location of the cluster.
  - If the event is within the cluster, add the event to the cluster by pushing the cluster a bit toward the event and updating the last event time of the cluster. The new cluster location ${\vec{x}}_{n+1}$ is given by mixing the predicted value of the old location $\left({\vec{x}}_{n}+\vec{v}\Delta t\right)$, where $\vec{v}$ is the cluster velocity and Δt is the time between this event and the last one that updated this cluster, with the event location $\vec{e}$using an adjustable mixing factor α≈ 0.01:
    $${\vec{x}}_{n\;+\;1}=(1-\alpha )\text{​}\left({\vec{x}}_{n}+v_{n}\Delta t\right)+\alpha \vec{e}$$
    This step implements a predictive tracker by giving clusters a kind of momentum that helps keep clusters attached to rapidly moving objects even if they emit few events. If the present event appears at the predicted location of the cluster, the clusters location is only modified to the predicted location. Events from the leading edge of the object pull the cluster forward and speed it up, while events at the cluster's trailing edge pull the cluster back and slow it down.
  - If the event is not in any cluster, seed a new cluster if there are spare unused clusters to allocate. The goalie typically uses 20 potential clusters.

A cluster is not marked as “visible” until it receives a certain number of events (typically 10 in the goalie) and is moving at a minimum speed (typically 20 pixels/s in the goalie).

The goalie robot determines the ball object as the cluster that will next hit the goal line, based on the cluster positions and velocities. The ball cluster's location and velocity measurement are used to position the servo to intercept the ball. If there is no threatening ball, the goalie relaxes.

Accurate and rapid measurement of cluster velocity is important in the goalie application because it allows forward prediction of the proper position of the arm. A number of algorithms for estimating cluster velocity were tried. Low-pass filtering the instantaneous cluster velocity estimates that come from the cluster movements caused by each event is cheap to compute, but was not optimal because the lowpass filter takes too long to settle to an accurate estimate. The method presently used is a “rolling” least squares linear regression on the cluster locations at the ends of the last N packets of events. This method is almost as cheap to compute because it only updates least-squares summary statistics by adding in the new location and removing the oldest location, and it instantaneously settles to an “optimal” estimate. A value of *N* = 10 computes velocity estimates over about the last 10–30 ms of ball location.

### Goalie self-calibration

In an earlier version of the goalie (Delbruck and Lichtsteiner, 2007) the arm position was specified by adjustable “offset” and “gain” parameters that mapped a motor command to a certain arm position. It was difficult to calibrate this goalie accurately and every time the aim of the vision sensor was adjusted or moved accidentally (the goal bounces around quite a bit due to the arm movements) laborious manual calibration had to be done again. The goalie arm was also not visible to the goalie and so there was no straightforward way for the goalie to calibrate itself. In the present goalie, the orientation of the arm was changed so that it swings on a horizontal plane rather than hanging like a pendulum and used a wide angle lens (3.6 mm) that allows the vision sensor to see both incoming balls and the goalie's hand. The horizontal arm orientation has the additional advantage that it allows the goalie to block corner shots much better.

**Figure 4:**
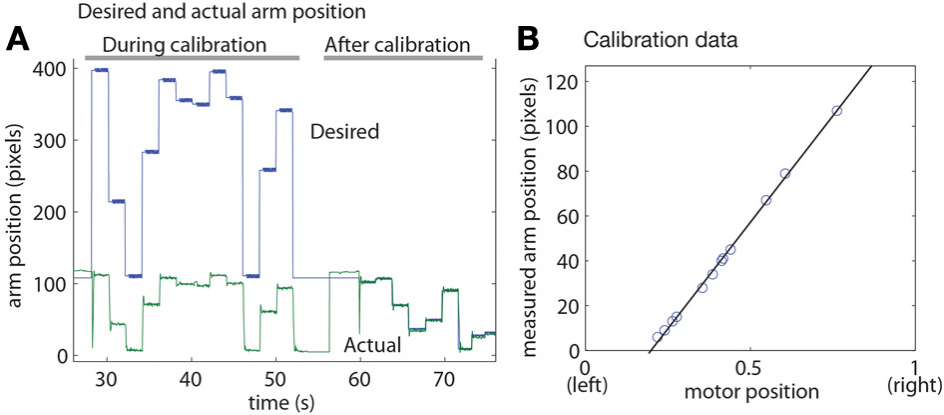
**Goalie self-calibration. (A)** Desired and actual arm position before and after calibration (the desired positions are the inverse mapping from motor commands in the allowed physical range to the pixel space). **(B)** Example measured arm positions vs. servo command used for least squares linear regression calibration.

Goalie calibration occurs in the learning state. When active, the arm position is tracked by using a motion tracker like the ball tracker but with a single cluster sized to the size and aspect ratio of the arm (Figure 3). The *x* position of the arm tracker is the arm coordinate in image space. The motor is controlled in coordinates chosen in software to span [0-1] units. The calibration algorithm has the following steps demonstrated by the data shown in Figure 4:

1. The present calibration is checked by randomly placing the arm in 5 pixel positions (using the current calibration parameters to determine the mapping) and measuring the actual arm position in pixel coordinates. If the average absolute error is smaller than a threshold (typically 5 pixels) calibration is finished. In the situation shown in Figure 4A, the calibration is initially very incorrect, and learning is initiated.
2. If calibration is needed, the algorithm places the arm in randomly chosen motor positions within a range specified in a GUI interface to be in roughly in the center of the field of view. (The GUI allows interactive determination of the servo motor limits). For each placement position, the actual pixel position is measured from the arm tracker. Typically 20 points are collected. A least-squares linear regression then determines the linear mapping from desired pixel position to motor position (Figure 4B). The algorithm then goes back to step 1. In Figure 4A, the calibration is checked after fitting and is satisfactory, so the calibration algorithms is terminated.

Calibration typically achieves accuracy within 2–5 pixels over the entire range. The linear approximation sin(*x*) = *x*near *x* = 0 was sufficiently accurate that it was not necessary to account for the sinusoidal relation between servo command and location of the arm across the goal.

### USB interfaces and servo control

Both the vision sensor and the servo controller use the Java interface provided by the Thesycon Windows USB driver development kit for Windows (www.thesycon.de). The servo commands are sent to the microcontroller in a separate writer thread that takes commands placed in a queue by the retina event processing thread. This decoupling allows for full speed USB 2.0 event processing although servo controller commands are transmitted using USB full-speed protocol at 12 Mbps (Axelson, 2001). The servo motor control command rate is 500 Hz, because each command requires 2 polls from the host controller and the minimal possible USB2.0 full-speed polling interval of 1 ms. The command queue length is set to one to minimize latency. New commands replace old ones if they have not yet been transmitted. Likewise, the incoming DVS events are transmitted in 128-event (or smaller) blocks and processed in a high priority thread that runs independently from the GUI or rendering threads. The DVS uses a USB2.0 high-speed interface with a data rate of 480 Mbps and a polling interval of 128 us. The USB interface threads were set to high priority, with highest priority given to the servo writing thread. Java's maximum priority is equivalent to Windows TIME_CRITICAL priority (Oaks and Wong, 2004).

A HiTec HS-6965 MG digital servo moves the goalie arm. This $120 hobby digital servo accepts pulse-width modulation (PWM) input up to at least the183 Hz frequency that we used and is rated to rotate 60° with no load in 100 ms. It can move the 40 cm long 20 g mass arm across the goal in about 100 ms and is slightly (~10%) underdamped with the goalie arm as only load. Other fast servos can be severely underdamped and actually oscillate (e.g., the Futaba S9253). The remaining overshoot with the HiTec servo is enough that the servo occasionally overshoots its intended location enough that the ball is not blocked.

A custom board based on the Silicon Labs C8051F320 USB2.0 full-speed microcontroller (www.silabs.com) interfaces between the PC and the servo motor. The microcontroller accepts commands over a USB bulk endpoint (Axelson, 2001) that program the PWM output width. The servo motor is powered directly by the 5V USB VBUS and 0.5F of ultracapacitor on the controller board helps to ballast the 5V USB VBUS voltage. The servo controller design is open-sourced in jAER (2007).

The servo arm is constructed from a paint stirrer stick with a balsa wood “hand” glued to its end. A goal of this project was to make this hand as small as possible to demonstrate the precision of tracking. The hand width used in this study was about 1.5 times the ball width (Figure 1).

## Results

Ordinarily a good shooter can aim most of the shots within the goal; thus a good shooter can potentially score on most shots. In a trial with several experienced shooters who were told they could take as much time as they needed to shoot, it required an average of 40 shots to score 10 goals. This means that each ball had to be shot about 4 times to score once, representing a shot success rate of 25%. A post experiment analysis of the data showed that the shooters could potentially have scored on 75% of their shots, with the rest of the shots representing misses wide of the goal (the shooters were intentionally aiming at the corners of the goal). Therefore, they had 30 shots on the goal and the goalie blocked 20 of these shots. The missed blocks consisted of a mixture of shots were not blocked for three reasons, ranked from highest to lowest occurrence: (1) they were so hard that they exceeded the ability of the servo to move the arm to the correct position in time; (2) tracking noise so that the arm position was not correctly computed well-enough; (3) servo overshoot, where the servo tries to move the arm to the correct position but because of the underdamped dynamics, the arm momentarily overshoots the correct position, allowing the ball to pass by.

**Figure 5:**
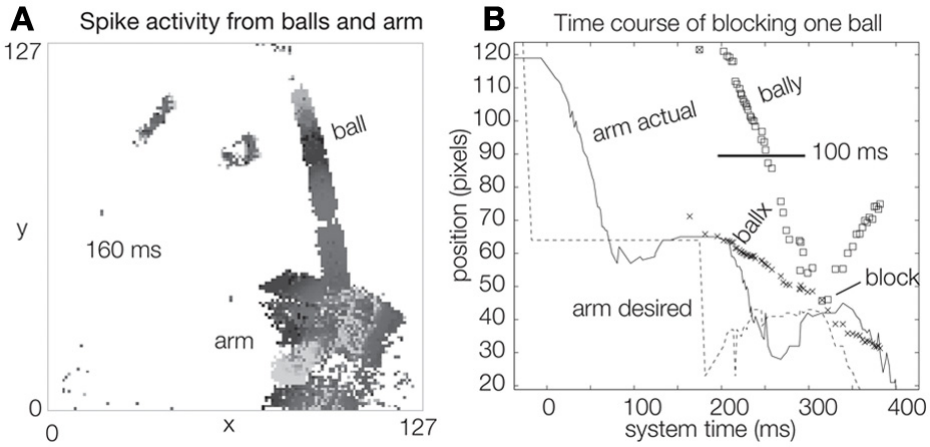
**Single shot dynamics. (A)** 2D histogram of spike activity caused by balls and goalie arm over 160 ms. **(B)** Time course of blocking one ball.

The cluster tracker algorithm is effective for ignoring distracters In Figure 3 four balls are simultaneously tracked. The topmost “ball” is probably the shooter's hand. Two balls are rolling away from the goal and are thus ignored. One is approaching the goal and the arm is moving to block it, based on the ball's position and velocity. Ignoring the many distracters would be impossible using a simpler method of ball tracking, such as median event location. Figure 5 shows the dynamics of a single blocking event for a ball that was shot quite fast, so that that it covers the distance from the top of the scene to the goal in about 100 ms. During the ball's 100 ms approach, about 50 packets of events, and thus samples of the ball position (“*ballx*” and “*bally*”), are captured by the tracker. The bounce off the arm is visible as the inflection in *bally*. The “desired arm position” is shown also as a function of time and is computed from *ballx, bally*, and the ball *x* and *y* velocities (not shown). The ball velocities are estimated by rolling linear regressions over the past 10 ball position samples for *ballx* and *bally* vs. time. The “actual arm position” is the position of the arm as measured by the arm tracker and it can be seen that the arm requires about 80 ms to move to the correct blocking position and also exhibits about 10% overshoot which is due to slight under-damping in the servo's controller. The response latency is dominated by the arm movement and the delay between knowing the desired arm position and the initiation of arm movement.

**Figure 6:**
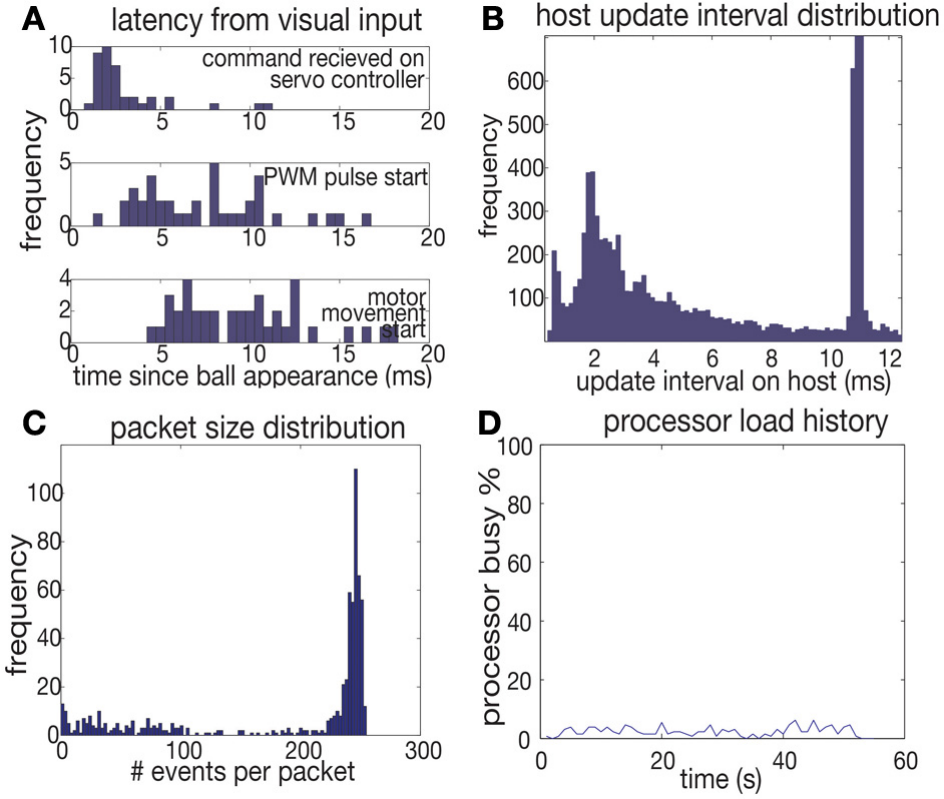
**Statistics. (A)** Latency measurements. Stimulus was flashing LED turned on at time 0. **(B)** Host packet processing interval distribution during normal operation while goalie is under attack. **(C)** Histogram of number of events per packet during normal operation. **(D)** Processor load during normal operation (2 balls/second attack).

Events are processed by the goalie software at a rate of 2 Meps (million events per second) on a 2.1 GHz Pentium M laptop running Windows XP, Java JVM version 1.6. During typical goalie operation, the average event rate is 20 keps, varying between <1 keps when idle to a maximum of 100 keps during active 10 ms windows of time. For buffers of 128 events processing the goalie code requires about 60 us. Figure 6B shows a histogram of processing intervals as recorded on the host PC using Java's *System.nanoTime()*. The median interval is 1.8 ms (the peak in the histogram at 10 ms is caused by forced transfers of data from the vision sensor at 100 Hz rate even when the USB FIFOs have not filled). During processing the computer's CPU load never rises over 4% (Figure 6D).

In this system sensor-to-computer latency is dominated by the USB FIFO filling time. The vision sensor pixel latency is inversely proportional to illumination (Lichtsteiner et al., 2007) and is about 100 us at normal indoor office illumination levels of 500 lux. A single ball that produces events at a peak rate of 100 keps causes a device-side 128-event USB packet about every 1 ms, although bursts of events can cause USB transfers that are received as often as every 128 us, the minimum USB2.0 high-speed polling interval. Increased retina activity (caused, say, by the arm movement) actually reduces this latency, but only because the USB device FIFO buffers are filled more rapidly. We used host side USB packet sizes of 256 events to match the maximum 500 Hz rate of writing commands to the servo motor, and the distribution of packet sizes reflects this (Figure 6C).

To measure latency, an artificial stimulus consisting of a flashing LED was set up so that it could be activated in bursts to mimic an instantaneously appearing ball. The servo controller was programmed to toggle an output pin when it received a servo motor command. The start of PWM output from the servo controller and the actual start of motor movement were measured. (The motor movement was measured from the power supply drop on the servo power supply). The measured median latency of 2.2 ms between the beginning of the LED flashing and the microcontroller output is the response latency leaving out the latency of the random PWM phase and the servo motor (Figure 6A). This latency was achieved by setting the servo controller USB2.0 full speed interrupt polling interval to 1 ms in the device's USB descriptor (Axelson, 2001); using the default polling interval of 10 ms resulted in substantially higher median latency of 5.5 ms that varied approximately bi-modally between 3 and 10 ms. The total latency for actuating the motor (5–15 ms) is dominated by the variable delay of PWM phase. The 183 Hz servo pulse frequency used in the robot has a period of 5.5 ms. A custom servo which directly accepted USB commands could reduce servo latency to about 1–2 ms, the delay to send a single USB1.1 command.

## Conclusion

The main achievement of this work is the concrete demonstration of a spike-event driven hybrid of a neuromorphic-sensor coupled to conventional procedural processing for low latency object tracking, sensory motor processing, and self-calibration. Secondary achievements are developments of robust and high speed event-based object tracking and velocity estimation algorithms. This paper also reports practical observations on the use of USB interfaces for sensors and actuators.

The goalie robot can successfully block balls even when these are low contrast white-on-gray objects and there are many background distracters. Running with standard USB buses for vision sensor input and servo-motor output under a standard preemptive multitasking operating system, this system achieves median update rates of 550 Hz, with latencies of 2.2 ± 2 ms from ball movement to motor command at a peak CPU load of less than 4%.

A comparable system based on using a standard image sensor would require a frame rate of at least 500 Hz. At the same spatial resolution (16 k pixels), a computer would need to continuously process 16 MBps of raw pixel information (with an 8-bit sensor output) to extract the basic visual information about changing pixels. Although this computation is certainly possible, the scaling to higher resolution is very unfavorable to the frame-based approach. Increasing the resolution to VGA resolution (640 × 480) at 1 kHz, for instance, would require processing 307 MBps, about 3 times the effective capacity of a high speed USB 2.0 interface and would allow only 3.3 ns per pixel of processing time. A VGA-sized DVS would generate about 18 times more data than the 128 × 128 sensor used for this paper if the objects filled a proportionally larger number of pixels, but even then the processing of the estimated 400 keps from the sensor would barely load a present-day's microprocessor CPU load and would be within the capabilities of modestly-powered embedded processors. As demonstrated by this work and other implementations (Linares-Barranco et al., 2007; Conradt et al., 2009a; Domínguez-Morales et al., 2012; Ni et al., 2013), the use of event-driven sensors can enable faster and lower-power robots of the future.

## Conflict of interest statement

The spinoff inilabs GmbH of the Inst. of Neuroinformatics is actively marketing dynamic vision sensor technology, selling vision sensor prototypes, and supporting users of the technology. The authors declare that the research was conducted in the absence of any commercial or financial relationships that could be construed as a potential conflict of interest.
